# Adjusted productivity costs of stroke by human capital and friction cost methods: a Northern Finland Birth Cohort 1966 study

**DOI:** 10.1007/s10198-021-01271-7

**Published:** 2021-02-24

**Authors:** Ina Rissanen, Leena Ala-Mursula, Iiro Nerg, Marko Korhonen

**Affiliations:** 1 Center for Life Course Health Research, University of Oulu, Oulu, Finland; 2 Medical Research Center Oulu, Oulu University Hospital and University of Oulu, Oulu, Finland; 3 Julius Center for Health Sciences and Primary Care, University Medical Center Utrecht and Utrecht University, Huispost nr. STR 6.131, P.O. Box 85500, 3508 GA Utrecht, The Netherlands; 4 Oulu Business School, Department of Economics, University of Oulu, Oulu, Finland

**Keywords:** Productivity costs, Human capital method, Friction cost method, Stroke, Cohort study

## Abstract

**Background:**

Productivity costs result from loss of paid and unpaid work and replacements due to morbidity and mortality. They are usually assessed in health economic evaluations with human capital method (HCM) or friction cost method (FCM). The methodology for estimating lost productivity is an area of considerable debate.

**Objective:**

To compare traditional and adjusted HCM and FCM productivity cost estimates among young stroke patients.

**Methods:**

The Northern Finland Birth Cohort 1966 was followed until the age of 50 to identify all 339 stroke patients whose productivity costs were estimated with traditional, occupation-specific and adjusted HCM and FCM models by using detailed, national register-based data on care, disability, mortality, education, taxation and labour market.

**Results:**

Compared to traditional HCM, taking into account occupational class, national unemployment rate, disability-free life expectancy and decline in work ability, the productivity cost estimate decreased by a third, from €255,960 to €166,050. When traditional FCM was adjusted for occupational class and national unemployment rate, the estimate more than doubled from €3,040 to €7,020. HCM was more sensitive to adjustments for discount rate and wage growth rate than FCM.

**Conclusions:**

This study highlights the importance of adjustments of HCM and FCM. Routine register-based data can be used for accurate productivity cost estimates of health shocks.

**Supplementary Information:**

The online version contains supplementary material available at 10.1007/s10198-021-01271-7.

## Introduction

Productivity costs are costs resulting from loss of paid and unpaid work and replacement costs due to morbidity and mortality. They are usually assessed in health economic evaluations with either human capital method (HCM) or friction cost method (FCM) [1–3]. Due to their major role in the total costs of illnesses, both overestimation and underestimation of productivity costs may lead to erroneous decision making [3]. The method for estimating lost productivity is an area of considerable debate [2–5]. Both HCM and FCM have their own purposes, seeking answers to different questions [6].

In HCM, the productivity costs are estimated as the value of lost productivity due to a disease over the expected remaining working life [1, 7]. HCM has been criticized for overestimating costs as it measures lost potential productivity instead of actual values, leading to unrealistically high estimates. HCM is based on the hidden assumption that, had the illness been avoided, the person would have remained alive, healthy and employed until the retirement age. However, it is common that people have several diseases and disabilities [8]. For example, people in a risk for stroke have increased work absence, risk of premature death, disability and unemployment even without the stroke event [9–12]. Also, HCM has been criticized for ignoring the possibility of employee replacement [13].

The FCM was developed to provide an alternative to HCM by focusing on organizational aspects and replacement issues [5, 13]. In FCM, the estimated costs accumulate only during the period an organization requires to restore its original production levels after losing a worker, i.e., a friction period [13]. The FCM usually assumes the friction period to be the average duration of vacancies increased with the expected time that employers need to place a vacancy and to train a replacing worker. Even though FCM was developed to offer a different perspective to productivity cost estimation, it does not overcome all HCM issues and leads to some shortcomings of its own. The major criticism against FCM has been that it does not consider the chain of vacancy filling resulting from already employed persons changing jobs [6].

Both HCM and FCM approaches share some limitations. One major limitation is that they do not take into account differences in wage levels between occupational classes. It has been highlighted in previous literature that the applied monetary values should resemble the value of lost productivity as closely as possible [14]. In both approaches, the conventional use of population mean wage might lead to overestimation of productivity costs if the disease burden were concentrated on lower socioeconomical classes. Previous studies of FCM have encouraged the use of different friction periods according to occupational classes since some occupations are less easy to replace [5, 13, 15, 16]. However, the friction period does not only depend on the type of occupation, but also the local employment circumstances [6].

It has been found that productivity cost estimates remarkably vary according to the choice of method in many disabling diseases such as stroke, schizophrenia and back pain [17–19]. The lack of consensus concerning which method to use, and what assumptions to make, is a source of ambiguity in productivity cost estimations. Researchers may end up choosing methods depending on whether the high or low productivity cost estimates suit their purposes better [7]. Altogether, there is a need for better understanding on the question-related feasibility and relevance of the competing methods to estimate productivity costs.

This paper highlights the divergences in both HCM and FCM methods based on their underpinning assumptions by using various adjusted models to compute productivity costs of stroke at young age. Stroke is the fourth most common cause of death in the European Union (EU) and even more significant cause of disability, leaving permanent disability to half of the survivors and causing the highest loss of quality-adjusted life years of all diseases [20–22]. In the EU, stroke hits every year more than 600,000 people, a quarter of whom are at working age [21] and the incidence of stroke among young persons (< 50 years) is increasing [23–26]. For these reasons, the economic burden of stroke is high, especially at the societal level. The total costs of stroke were estimated 57 billion euros in the EU in 2017 [27], and they are expected to rise [21]. It has been estimated that productivity costs, i.e., costs due to loss of paid and unpaid work, constitute more than half of total lifetime costs per stroke patient in the EU [20]. The productivity costs due to premature death and early retirement are highest among the working-aged, among which the increasing incidence of strokes has raised urgency to evaluate the consequent productivity costs.

Aim of this study was to compare estimates of productivity costs by using both traditional and adjusted HCM and FCM models among young stroke patients. In this study, we (1) use detailed, accurate register-based data of a large population-based birth cohort, (2) control for occupation-specific costs, macroeconomic conditions, vacancy chains and disability conditions on the estimation of productivity costs, (3) apply state-of-the-art productivity cost analyses based on adjusted productivity cost methods.

## Materials and methods

### Setting

We conducted three different model specifications of both HCM and FCM cost estimates in a Finnish stroke population: base models, occupation-specific models and adjusted models. To estimate productivity costs using these models, we linked personal level data on incident strokes, work absence, mortality and income with population level data as detailed below and in Table 1. We estimated the productivity costs separately in different stroke subtypes: ischemic strokes (IS), transient ischemic attacks (TIA), haemorrhagic strokes (HS) (including subarachnoid haemorrhages and intracerebral haemorrhages) and unspecified strokes (US). Sensitivity analyses were conducted to demonstrate the robustness of the models. All monetary values are presented in 2018 value, i.e., costs not available for 2018 were inflated to 2018 [28, 29]. We used the consumer price index (CPI) as a converter. All analyses were done in R version 4.0.3 [30].

**Table 1:** Data collection and use in HCM and FCM models

Variable	Available data	Used for	Calculated as	Data level	Data source	Available	Other notes	Link
Stroke diagnoses	Diagnostic code, hospitalization, date of stroke onset	Study population	n.a	Individual level data	Hospital discharge register; ICD-10 codes I60-69 and G45 and ICD-9 codes 430–438	1967–2015	Available until age of 50 years	1, 2
Mortality	Cause and date of death	Absent days after the stroke event	n.a	Individual level data	Statistics Finland	1967–2015	n.a	3
Sick leaves and disability pensions	Dates of sick leaves longer than 10 days, disability pensions	Absent days before and after the stroke event	n.a	Individual level data	SII and FCP	1970–2016	Absent days before 18th birthday were not included	4, 5
Gross income	Annual gross income	Monetary value of production	Daily wage = annual wage/365 (366 for leap years)	Individual level data used to calculate median of study population	Finnish Tax Administration	1995–2016	Discounted to 2018 using monetary value factor	6
Monetary value factor	Value of money	Income in 2018 value	n.a	Population level data	Statistics Finland	1860–2018	Before 1995 and after 2016 annual constant rate of 3% was used	7,8
GDP growth rate	Annual GPD growth	Proxy for annual wage growth before 1995 and after 2016	n.a	Population level data	World Development Indicators, The World Bank and Statistics Finland	1961–2019	Annual 1.79% growth rate (mean of 2015–2019) used after 2019	9
Occupational class	Annual occupational class	Occupation specific calculations	n.a	Individual level data	Statistics Finland	1995, 2000, 2004–2014	1995 value used for 1984–1999, 2000 value used for 2000–2003	10
Disability free life expectancy	Disability pensions	Adjusted HCM	% of same aged population on disability pension	Population level data	Finnish Institute for Health and Welfare	1996–2018	Data available for age group 25–64 was used in calculations	11, 12
Unemployment rate (u)	Annual % unemployed, % underemployed, and % hidden unemployed for both genders	Adjusted HCM and LVC calculations	Unemployed + hidden unemployed + ½ *underemployed	Population level data	Statistics Finland	1997–2019	Data was extended by using the mean of first/last five observations of the relevant age group	13, 14
Life expectancy	For both genders and ages between 18–65	PLDF (disability free life expectancy/life expectancy)	Estimated after 2018 by the projection in year 2020 for ages 53–56, 2025 for ages 57–61, 2030 for ages 62–65	Population level data	Statistics Finland	1986–2018, projection for 2020, 2025, 2030, 2035	Expanded by using the first observation for appropriate age. Projection based on 2019 prediction	15–18
Proportion of employed jobseekers (α)	Jobseekers, unemployed jobseekers; separately for occupational classes	LVC calculations	Number of jobseekers minus number of unemployed jobseekers	Population level data	Statistics Finland	2006–2019	Data extended by using the mean of 5 first/last observations each gender and occupational group, using the appropriate age group	19
Work ability (WA)	Self-reported work ability	Adjusted HCM	Mean of individual answers	Individual level data used to calculate population level index	Questionnaires to NFBC1966 cohort: “How would you score your current work ability as compared to lifetime best?” scaled from 0 (no work ability) to 10 (at maximum)	1997 and 2012	One occupational class specific index was used for all years	
Work ability decline (WAD)	WA from NFBC1966 questionnaires	Adjusted HCM	1—(WA in year 1997–WA in year 2012)/(WA in year 1997)	Individual level data used to calculate population level index	Questionnaires to NFBC1966 cohort	1997 and 2012	To avoid zero values as denominator, we added + 1 for WA in year 1997 and 2012	
Occupational vacancy period	Annual average vacancy of filled positions for each occupational class	Occupation specific and adjusted FCM	n.a	Population level data	Ministry of Economic Affairs and Employment and Statistics Finland	2006–2018	Average of 2006–2009 used as an estimate for 1984–2005, and average of 2015–2018 used as an estimate for 2019 onwards	20, 21
Occupational friction period	Average occupation-specific vacancy period increased with 60 days	Occupation specific and adjusted FCM	Occupational vacancy period + 60 days	Population level data	Ministry of Economic Affairs and Employment and Statistics Finland	1984 onwards		
Length of vacancy chain (LVC)	α and u presented previously	Adjusted FCM	α × (1 − u)/u	Population level data	Statistics Finland	1984 onwards		

*ICD* International Classification of Diseases, *SII* Social Insurance Institution, *FCP* Finnish Center for Pensions, *GDP* Gross Domestic Product, *n.a.* not applicable, links:

https://www.who.int/classifications/icd/icdonlineversions/en/

https://apps.who.int/iris/handle/10665/39473

https://www.stat.fi/index_en.html

https://www.kela.fi/web/en

https://www.etk.fi/en/

https://www.vero.fi/en

https://www.stat.fi/til/khi/2018/khi_2018_2019-01-22_tau_001.html

https://www.stat.fi/tup/laskurit/rahanarvonmuunnin_en.html

https://data.worldbank.org/indicator/NY.GDP.MKTP.KD.ZG

https://www.tilastokeskus.fi/fi/luokitukset/ammatti/

https://sotkanet.fi/sotkanet/en/metadata/indicators/306

https://sotkanet.fi/sotkanet/en/taulukko/?indicator=szbxBwA=&region=s07MBAA=&year=sy5ztC7V0zUEAA==&gender=m;f&abs=f&color=f&buildVersion=3.0

https://www.stat.fi/til/tyti/index.html

http://pxnet2.stat.fi/PXWeb/pxweb/fi/StatFin/StatFin__tym__tyti__vv/statfin_tyti_pxt_11pp.px/

http://www.stat.fi/til/kuol/meta.html

http://pxnet2.stat.fi/PXWeb/pxweb/fi/StatFin/StatFin__vrm__kuol/statfin_kuol_pxt_12ap.px/table/tableViewLayout1/

http://www.stat.fi/til/vaenn/meta.html

http://pxnet2.stat.fi/PXWeb/pxweb/fi/StatFin/StatFin__vrm__vaenn/statfin_vaenn_pxt_129b.px/

http://pxnet2.stat.fi/PXWeb/pxweb/en/StatFin/StatFin__tym__tyonv__vv/statfin_tyonv_pxt_1510.px/

http://www.stat.fi/til/tyonv/index_en.html

http://pxnet2.stat.fi/PXWeb/pxweb/en/StatFin/StatFin__tym__tyonv__vv/statfin_tyonv_pxt_2510.px/

### Individual level data collection

The Northern Finland Birth Cohort 1966 (NFBC1966) is a non-selective, prospective, population-based birth cohort followed-up since the mid-pregnancy [31, 32]. The cohort is based on 12,058 alive-born children whose expected birth date was in 1966, representing 96% of children born in Finnish Provinces of Lapland and Oulu in 1966.

From the entire cohort, all those who had a stroke before age of 50 years were selected into this study population, consisting of 339 stroke patients (2.8% of original cohort population). By the time of data collection, information on stroke diagnoses and hospitalizations was available only until the end of year 2015, i.e. the 50th year of cohort members’ life. Diagnoses and hospitalizations were obtained from national hospital discharge registers, from which the subjects with International Classification of Diseases (ICD) codes I60–69 and G45 from ICD-10 [33], and codes 430–438 from ICD-9 [34] were identified. The individual data included also the date of the stroke onset.

Individual level data of death notes, including cause and date of death, were collected from Statistics Finland [35] until the end of 2015. Data on sick leaves and disability pensions were collected from the registers of Social Insurance Institution (SII) [36] and Finnish Center for Pensions (FCP) [37]. The data of SII included the dates of sick leaves longer than 10 days and non-earnings-based disability pensions between years 1970 and 2016. Earnings-based pensions were collected from FCP until the year 2016. In Finland, pension security covers all paid work, also in self-employment. Sick leaves with length of 10 days or less are not present in these registers because that period is not included in the sickness allowance covered by social insurance. The healthcare registers and sickness allowance systems of Finland are described in the Supplement.

The length of work absence before and after the stroke event was calculated for each stroke patient based on starting and ending dates of sickness leaves, fixed-term disability pensions, permanent disability pensions, death dates and the stroke dates. The data on sick leaves, disability pensions and deaths were used to assign the absence from work from 18th birthday until the end of 2016. Even if the stroke occurred before 18th birthday, absent days before that were not included in productivity cost estimations because productivity costs were assumed to cumulate only when patients were in their working age (18–65 years). If person had died or had been granted a permanent disability pension by the end of 2016, the person was assumed to be absent from work until their retirement age (65 years). The sickness periods shorter than 10 days, which are not covered by the registers, were corrected in the FCM by adding 10 absent days or the number of days between stroke diagnosis and the start of sick leave, to the number of absent days from work. This correction was not included in HCM.

The register of The Finnish Tax Administration [38] was used to obtain individual level data on the gross income of cohort members. The annual gross income data were available from 1995 to 2016. Daily wage was calculated from personal annual gross income by dividing it by 365 (366 for leap years). Median daily wage of the NFBC1966 cohort population available from 1995 to 2016 was used in calculations. The value of past and future daily productivity was discounted at a constant annual rate of 3.0% recommended in Finnish guidelines [39] to the reference year 2018. We used the CPI as a converter. We used the annual Gross Domestic Product growth rates as a proxy for wage growth before 1995 and for period 2017–2019. An annual growth rate of 1.79% was used to predict annual wages after 2019, based on the Finnish annual economic growth rate between 2015 and 2019.

Information on perceived work ability (WA) was obtained during examinations of NFBC1966 members in 1997 (*n* = 8 767) and 2012 (*n* = 6 774), with a survey question *“How would you score your current work ability as compared to lifetime best?”* scaled from 0 (no work ability) to 10 (at maximum). Instead of using individual values in cost estimates, we calculated an index of overall work ability decline (WAD) in study population.

### Population level data collection

The population level annual data on average disability free life expectancy were obtained from the Finnish Institute for Health and Welfare open access materials [40]. The disability free life expectancy was estimated as the % of total population of same age receiving disability pension and the annual data were available from 1996 to 2018. The population level data for the unemployment rate and for life expectancy were obtained from Statistics Finland open access materials [41–43]. The annual unemployment rates were available from 1997 to 2019 and the annual life expectancy data from 1986 to 2018 including projections for life expectancy from 2020 onward.

The annual population level data on vacancy periods according to occupational classes were obtained from official statistics of Ministry of Economic Affairs and Employment between 2006 and 2018 [44]. Information on proportion of employed jobseekers was estimated from the open-source material provided by Statistics Finland [45], defined as number of jobseekers minus number of unemployed jobseekers divided by total number of jobseekers. The annual population level data are available from 2006 to 2019.

### HCM

In HCM, the productivity costs were calculated as days absent from work after a stroke because of sick leave, disability pension or death until the statutory retirement age, multiplied with the value of daily production each year. We used three different approximations for the value.

In the base HCM, the median wage of total NFBC 1966 population, stratified by gender, was used to monetarize the value of production, regardless of differences in actual personal wages. In the occupation-specific HCM, the median wage of each occupational class was used, again stratified by sex. In the adjusted HCM, we further adjusted occupation-specific values for the future labour force participation rates by taking into account proportion of disability-free life expectancy (PLDF) and unemployment coefficients and overall coefficient of work ability decline. These adjustments were based on the study of Targoutzidis [6]. More specifically, we estimated the productivity costs (PC) as

$${\text{PC}} = {\text{wage}} \times t \times {\text{PLDF}} \times \left( {1 \, - \, u} \right) \times {\text{WAD}},$$

where *t* is the time of absence (until the retirement age), PLDF = disability-free life expectancy/life expectancy, *u* is the national annual unemployment rate and *WAD* is an estimate for overall coefficient of work ability decline.

### FCM

In FCM, the productivity costs were evaluated as length of the absence from work after a stroke multiplied with the value of daily production if absence was shorter than estimated friction period. However, if the length of one’s absence from work was longer than the friction period, the friction period was used in calculation instead of length of actual absence. We formed three separate models to estimate the productivity costs.

In the base FCM model, the length of friction period was assumed to be 60 days, the time employers need to place a vacancy and to train replacement of absent worker, based on previous international literature [15, 46]. In this model the vacancy was assumed to be filled immediately after it was placed. The median wage of the total NFBC1966 population was used to monetarize the value of production in the base FCM model. In the occupation-specific FCM model, separate median wages and friction periods were used for occupational classes instead of the population median. The friction period was annually estimated to be the occupation-specific average vacancy period increased with 60 days that was assumed to be the time employers need to place a vacancy and to train replacement of absent worker.

In the adjusted FCM model, we took into account the length of vacancy chain (LVC) as suggested by Targoutzidis [6]. We did this further adjustment to the occupation-specific FCM model. A simple estimate for the length of the vacancy chain (LVC) is

$${\text{LVC}} = \alpha \times \left( {1 - u} \right)/u,$$

where *u* is the annual unemployment rate and *α* is the proportion of employed jobseekers.

### Sensitivity analyses

We conducted various sensitivity analyses for our results. For all models, we varied wage growth and discount rates. Wage growth rates 0% and 4% were applied to establish the sensitivity of estimate to labour productivity. The discount rates were varied from 0 to 5%. We also conducted analyses where we altered the unemployment rate (*u*) between minimum and maximum observed rates. In addition to these, the friction period was varied from 60 to 180 days in FCM models, based on minimum and maximum friction periods as used in previous literature [15].

## Results

### Characteristics of stroke sample

During the follow-up from birth to age 50 years, 339 subjects (2.8% of total cohort) got a stroke. Of all strokes, 113 (33.3%) were IS, 108 (31.9%) were TIA, 85 (25.1%) were HS (59 subarachnoid haemorrhages and 26 intracerebral haemorrhages) and 33 (9.7%) were US (Table 2). The median age of stroke onset was lowest in HS and highest in TIA.

**Table 2:** Characteristics of stroke sample

	Total (*n* = 339)	IS (*n* = 113)	TIA (*n* = 108)	HS (*n* = 85)	US (*n* = 33)
	N (%)/Median (IQR)	N (%)/Median (IQR)	N (%)/Median (IQR)	N (%)/Median (IQR)	N (%)/Median (IQR)
Gender					
Male	172 (50.7%)	61 (54.0%)	48 (44.4%)	46 (54.1%)	17 (51.5%)
Female	167 (49.3%)	52 (46.0%)	60 (55.6%)	39 (45.9%)	16 (48.5%)
Occupation at the time of stroke					
Military personnel	5 (1.5%)	3 (2.7%)	2 (1.9%)	0 (0.0%)	0 (0.0%)
Managers	13 (3.8%)	1 (0.9%)	6 (5.6%)	3 (3.5%)	3 (9.1%)
Professionals	44 (13.0%)	11 (9.7%)	20 (18.5%)	9 (10.6%)	4 (12.1%)
Technicians and associate professionals	52 (15.3%)	17 (15.0%)	21 (19.4%)	9 (10.6%)	5 (15.2%)
Clerical support workers	23 (6.8%)	7 (6.2%)	7 (6.5%)	6 (7.1%)	3 (9.1%)
Service and sales workers	60 (17.7%)	21 (18.6%)	16 (14.8%)	16 (18.8%)	7 (21.2%)
Skilled agricultural, forestry and fishery workers	8 (2.4%)	5 (4.4%)	1 (0.9%)	1 (1.2%)	1 (3.0%)
Craft and related trades workers	34 (10.0%)	13 (11.5%)	11 (10.2%)	9 (10.6%)	1 (3.0%)
Plant and machine operators, and assemblers	33 (9.7%)	10 (8.8%)	11 (10.2%)	7 (8.2%)	5 (15.2%)
Elementary occupations	22 (6.5%)	4 (3.5%)	6 (5.6%)	10 (11.8%)	2 (6.1%)
Unknown	45 (13.3%)	21 (18.6%)	7 (6.5%)	15 (17.6%)	2 (6.1%)
Education					
Primary school or lower	39 (11.5%)	16 (14.2%)	8 (7.4%)	12 (14.1%)	3 (9.1%)
Upper secondary education	183 (54.0%)	62 (54.9%)	51 (47.2%)	54 (63.5%)	16 (48.5%)
Tertiary education	117 (34.5%)	35 (31.0%)	49 (45.4%)	19 (22.4%)	14 (42.4%)
Work status at the onset of stroke					
Working	129 (38.1%)	39 (34.5%)	54 (50.0%)	23 (27.1%)	13 (39.4%)
Absent due to sickness	89 (26.3%)	32 (28.3%)	25 (23.1%)	22 (25.9%)	10 (30.3%)
Unemployed	121 (35.7%)	42 (37.2%)	29 (29.9%)	40 (47.1%)	10 (30.3%)
Return to work force					
No	185 (54.6%)	71 (62.8%)	47 (43.5%)	53 (62.4%)	14 (42.4%)
Yes	154 (45.4%)	42 (37.2%)	61 (56.5%)	32 (37.6%)	19 (57.6%)
Mortality	38 (11.2%)	12 (10.6%)	1 (0.9%)	24 (28.2%)	1 (3.0%)
Median age at stroke onset	43.7 (IQR 8.6)	43.1 (IQR 8.3)	45.6 (IQR 6.2)	41.7 (IQR 12.3)	42.1 (IQR 12.5)
Median number of days off					
Before stroke	46 (IQR 379)	46 (IQR 686)	70 (IQR 360)	18 (IQR 91)	23 (IQR 1166)
After stroke	259 (IQR 6832)	514 (IQR 7357)	19 (IQR 587)	6410 (IQR 8464)	93 (IQR 5812)

*IS* ischemic stroke, *TIA* transient ischemic attack, *HS* hemorrhagic stroke, *US* unspecified stroke, *IQR* interquartile range

At the time of stroke event, 89 (26.3%) subjects were already on a sick leave or disability pension. After the stroke event, 38 (11.2%) subjects died until end of 2015. During the follow-up, 240 (70.8%) stroke patients had an absence from work, and 154 (45.4%) of stroke patients returned to work force. The median amount of absent days after stroke was 259 (interquartile range 6832) days in all strokes being lowest in TIA and highest in HS.

### Productivity costs

The average lifetime productivity costs per one stroke patient estimated with HCM and FCM base models, occupation-specific models and adjusted models, are shown in Table 3. Using the base HCM the productivity costs were €255,960 per patient. When wage differences in occupational classes were taken into account, the average productivity costs per one stroke patient were €212,890. With additional adjustments for disability-free years, unemployment and decline in work ability, the average productivity costs per one stroke patient were €170,030.

**Table 3:** Estimated productivity losses per person with three different HCM and FCM models in 2018 Euros

	HCM costs	FCM costs
Base model	€255,960	€3,040
Occupation-specific model	€212,890	€3,890
Adjusted model	€170,030	€7,200

*HCM* human capital method, *FCM* friction cost method

The average productivity costs per stroke patient were €3,040 when using the base FCM. When both the differences in wages and friction periods according to occupational classes were taken into account, the average productivity costs were €3,890. With additional adjustment for the length of the vacancy chain, the average productivity costs per one stroke patients rose to €7,200.

Average productivity costs by adjusted HCM and FCM models were highest in HS and lowest in TIA (Table 4). In adjusted HCM model the average productivity costs were higher among men, whereas in adjusted FCM they were higher among women. Concerning the age of stroke onset, the patients under 40 years had higher productivity costs with adjusted HCM than patients over 40 years, but lower costs when estimated with adjusted FCM. The higher educational level associated with lower productivity costs with adjusted HCM, but higher costs with adjusted FCM.

**Table 4:** Adjusted HCM and FCM estimates shown according to clinical and sociodemographic characteristics at stroke onset

	HCM costs	FCM costs
Gender		
Male (*n* = 172)	€171,440	€6,150
Female (*n* = 167)	€160,500	€7,920
Age at onset		
Under 40 (*n* = 108)	€203,990	€5,150
Over 40 (*n* = 231)	€148,310	€7,900
Education		
Primary school (*n* = 39)	€205,470	€4,890
Upper secondary education (*n* = 183)	€163,950	€6,570
Tertiary education (*n* = 117)	€156,190	€8,430
Occupation		
Military personnel (*n* = 5)	€4,500	€6,970
Managers (*n* = 13)	€69,080	€14,050
Professionals (*n* = 44)	€178,230	€9,550
Technicians and associate professionals (*n* = 52)	€171,420	€7,240
Clerical support workers (*n* = 23)	€191,400	€9,570
Service and sales workers (*n* = 60)	€173,770	€8,510
Skilled agricultural, forestry and fishery workers (*n* = 8)	€212,150	€4,730
Craft and related trades workers (*n* = 34)	€161,700	€4,580
Plant and machine operators, and assemblers (*n* = 33)	€181,210	€6,540
Elementary occupations (*n* = 22)	€172,270	€5,870
Unknown (*n* = 45)	€181,530	€3,460
Work status at the onset of stroke		
Working (*n* = 129)	€61,760	€6,380
Absent due to sickness or disability (*n* = 89)	€340,500	€10,370
Unemployed (*n* = 121)	€148,920	€5,240
Return to work force after stroke		
No (*n* = 185)	€222,400	€7,360
Yes (*n* = 154)	€98,360	€6,620
Stroke type		
IS (*n* = 113)	€184,950	€7,820
TIA (*n* = 108)	€81,430	€5,110
HS (*n* = 85)	€253,250	€8,420
US (*n* = 33)	€153,690	€6,950

*HCM* human capital method, *FCM* friction cost method, *IS* ischemic stroke, *TIA* transient ischemic attack, *HS* hemorrhagic stroke, *US* unspecified stroke

**Fig. 1:**
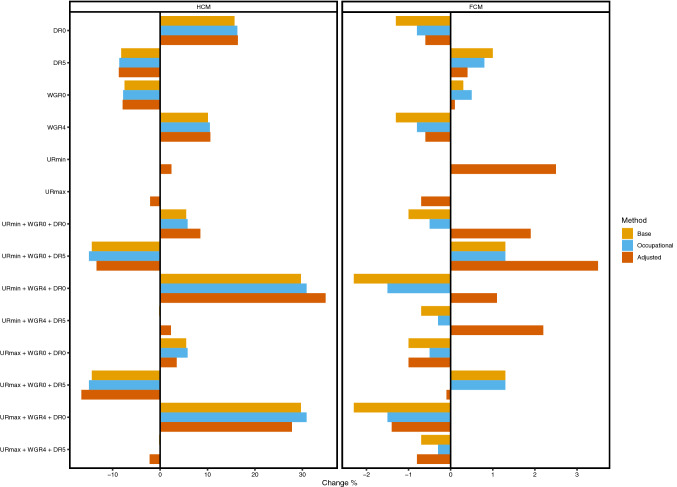
Results of sensitivity analyses of altering discount rates, wage growth rates, and unemployment rates. *HCM* human capital method, *FCM* friction cost method, *DR0* discount rate 0%, *DR5* discount rate 5%, *WGR0* wage growth rate 0%, *WGR4* wage growth rate 4%, *URmin* minimum unemployment rate, *URmax* maximal unemployment rate

**Fig. 2:**
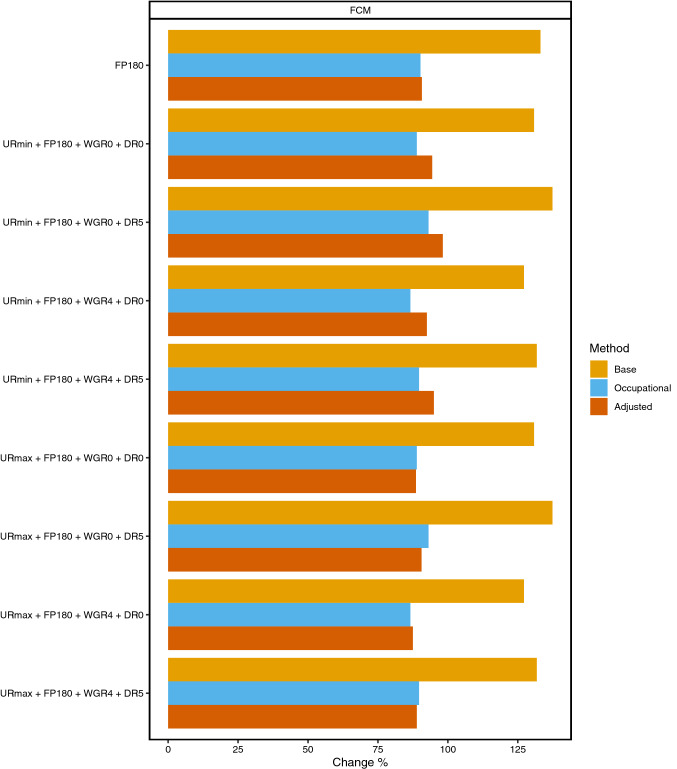
Results of sensitivity analyses of altering friction periods. *FCM* friction cost method, *FP180* friction period 180 days, *DR0* discount rate 0%, *DR5* discount rate 5%, *WGR0* wage growth rate 0%, *WGR4* wage growth rate 4%, *URmin* minimum unemployment rate, *URmax* maximal unemployment rate

Results of the sensitivity analyses are presented in Figs. 1 and 2. Variations in discount rates and wage growth rates altered the base HCM estimation between − 14.4% and + 29.7%. Occupation-specific HCM model altered between − 15.0% and + 30.9% and adjusted HCM model between − 15.1% and + 31.1%. In FCM sensitivity analyses, the base model altered between − 2.0% and + 1.6%, the occupation specific model between − 1.5% and + 1.3% and the adjusted model between − 1.0% and + 0.6%. In all models, the productivity costs estimates were lowest when discount rate was highest and wage growth rate was lowest and vice versa. Variation in unemployment rate altered the adjusted HCM model between − 2.1% and 2.4% and adjusted FCM model between − 0.7% and 2.5%.

Changing the maximum friction period from 60 to 180 days increased the values of base FCM model by 133.6%, occupation-specific FCM model by 90.2% and adjusted FCM model by 90.7% (Fig. 2). Variation in discount rate or in wage growth rate did not cause substantial additional changes on these estimates.

## Discussion

To our knowledge this is the first study to demonstrate in real-life setting how HCM and FCM productivity cost estimates change when several hidden assumptions in these methods as well as macroeconomic trends are taken into account. While we do not provide new methods to estimate productivity costs, our novel study design uses individual level data of a non-selective birth cohort to demonstrate the importance of adjustments when estimating productivity costs.

### Comparison of HCM and FCM

In this study, HCM provided considerably higher productivity cost estimates than FCM. Previously, variation in HCM and FCM estimates has depended on research questions and the studied diseases [7]. Studies assessing short-term sick leaves have found only small differences between the two approaches, whereas studies including productivity costs of mortality and permanent disability have found higher variation [18, 19, 47]. Diseases with low mortality and disability rates or with clear time-restricted courses of disease provide lower differences between HCM and FCM than severe diseases like stroke [7]. A previous study about productivity costs of another cardiovascular disease, coronary heart disease, found that high early retirement rates lead HCM to give more than hundred times higher morbidity cost estimates than FCM [47]. The fact that stroke is a severe condition causing permanent disability or death to many patients explains the high difference between HCM and FCM estimates found here. The choice of friction period plays also a role when comparing HCM and FCM estimates [14]. The longer the friction period, the closer to HCM estimates the results get.

We found that HCM was more sensitive to changes in discount rate and wage growth rate than FCM. In all FCM models, longer maximum friction periods considerably increased productivity cost estimates.

### Future participation in labour force

We adjusted HCM for the hidden assumption that by having had avoided stroke, the person would have remained alive, healthy and employed until the retirement age. This was done in adjusted model by taking into account the susceptibility to death and disability due to other conditions than stroke and the susceptibility to unemployment. We also took into account the wage differences in occupational classes instead of using a median wage of the total population, as stroke incidence, mortality and disability are not similar in occupational classes, and as using a population median might overestimate productivity costs. We found that when compared to the traditional way of estimating productivity costs with HCM, by taking into account occupational wage differences, national unemployment rate, proportion of disability free life expectancy, and overall decline in working ability, the estimate decreased by 35% from €255,960 to €166,050.

### Vacancy chain and labour market changes

In this study, FCM was adjusted for the hidden assumption that vacancy created by the disability of a worker will be filled by a previously unemployed person. The adjustment for vacancy chain was done by including the proportion of already employed jobseekers together with national unemployment rate in the adjusted model. We also considered the differences in wages and friction periods between the occupational classes. When the traditional FCM model was adjusted for differences in occupational classes and for the vacancy chain assumption, the estimate increased by 131% from €3,040 to €7,020.

A previous study has showed that labour market changes, in particular unemployment rate changes, have major effects on FCM productivity cost estimates [48]. However, in our sensitivity analyses, variation in unemployment rate did not notably alter the adjusted FCM estimates. Furthermore, our results show that some occupational categories markedly affected recruitment periods and prolonged the time to fill vacancies. This confirms the previous findings that the current practice of using only one pre-determined friction period for labour market circumstances leads to imprecise estimates for productivity costs [15]. It should be noted that we considered a friction period also in short-term absences even though there probably was no vacancy created.

### Workplace compensation

Health economists have paid attention to the existence of compensation mechanisms in workplaces, but it seems that there are contradictory ideas of these [4, 7, 13, 14] [49–52]. Therefore, in this study we decided not to take into account wage multipliers or elasticity of labour time productivity. According to economic theory, the gross wage is an appropriate measure of productivity, because companies continue to employ labour until the marginal cost of labour is equal to the marginal revenue. Our models are based on the assumption that the gross wage is the best estimate of monetary value of production. It should be mentioned that our study population was obtained from a birth cohort eliminating the effect of age on the value of production found in previous studies [53].

### Employment and occupation-related socioeconomic status

Productivity loss estimates due to the disease should capture not only the loss due to the disease itself but also the impact of its co-morbidities and the treatment effects on productivity are concerned. In this study we did not investigate work absence due to stroke, but work absence in stroke population. We found that only 38% of our study population were working at the time of stroke event. Of our population, 26% were already absent due to sickness or disability and 36% were unemployed. Those who were already absent before the stroke event, had higher productivity cost estimates than those who were working at the time of stroke. This highlights that studying costs of one disease is highly difficult, and previous diseases and co-morbidities have huge effect on work ability and productivity cost estimates. Also, previously unemployed had higher HCM estimates than previously working people. This is in line with previous literature showing that previous unemployment is associated with poor prognosis after stroke [54].

Our results showed that the higher the educational level, the higher the adjusted FCM cost estimates. In contrast, adjusted HCM estimates got lower when educational level increased. The same phenomenon was found regarding occupational classes as managers had the lowest HCM estimates and the highest FCM estimates. Previous studies have likewise showed that productivity cost estimates vary by socioeconomic positions [55]. These findings highlight the fact that HCM and FCM measure productivity costs from different perspectives. The results suggest that individuals with higher education and socioeconomic class are more likely to return to work after stroke than individuals with lower education and socioeconomic class. However, since this return to work does not happen inside the friction period, the wage differences override the absence length differences targeted in FCM. It can be postulated that in case of a severe medical disease such as stroke, FCM does not capture the differences in absence lengths between socioeconomic classes even when adjusted for occupation-specific friction periods.

### Equity concerns

Some previous studies have debated over the use of average wage rate of a population for generalizability and equity reasons [14, 56]. In this study we used gender- and occupational class specific median wages to monetarize the production instead of individual wages. Use of individual wages might encourage inequities in health care when labour market discrepancies are transposed to productivity cost estimates and economic evaluations suggest prioritizing health interventions unequally [14, 57]. All productivity cost estimates based on wages might then value the health of high-income earners over that of low-income earners, of workers over non-workers and of men over women. We showed that using gender-specific median wages, males had on average higher productivity costs in adjusted HCM model, but lower in adjusted FCM model.

In this study, productivity costs of previously non-employed stroke patients were also estimated to tackle equity concerns. Although there may be a practical justification for excluding productivity losses of non-workers, doing so would probably result in higher valuation of treatment aimed at working people. Such an approach would be hard to justify as equitable. We found that productivity cost estimates, especially HCM estimates, were higher among those previously absent from work due to sickness, disability or unemployment, compared to previously working individuals.

When using the FCM approach, the impact on equity considerations is complex, even if average wage rates were used to value production losses, because the estimates of production loss are partly driven by the duration of the friction period. The friction period will vary by job and by industry but is likely to be shorter for jobs performed by low-paid workers, because such workers tend to be easier to replace. In our study, managers had three times higher FCM productivity cost estimates than, *e.g*., agricultural or craft workers.

It has been previously suggested that productivity loss should be reported only in physical units, or that only average wages should be used to prevent equity concerns [55]. Our results show that medians of absent days after stroke vary between stroke subtypes more than the productivity cost estimates vary between them. This is in line with previous literature showing that neither HCM nor FCM cost estimates are equivalent to days lost [55]. One explanation to our findings might be reverse causality: patients with early-life stroke have not had the abilities needed to gain education or high employment status because of their severe disability. Overall, equity considerations should be taken into account in all health care policy making [58].

### Value of non-market production and presenteeism

In this study, the productivity cost estimates were restricted to lost paid employment. No value for nonmarket production, *e.g.,* informal care and unpaid volunteer work, was estimated. This may have caused underestimation of productivity costs. A recent European study found that of total productivity costs during the first year after a stroke, 18% were due to caregiver time loss [59]. Furthermore, the reduced productivity while at work, presenteeism, was not accounted for. This was inevitable because only objective register data were used in this study and no objective measures are available for presenteeism.

### Strengths and limitations

As the main strength of this study, our data allow us to demonstrate more accurate estimates of productivity costs among young stroke patients compared to previous studies. Importantly, we were able to adjust HCM and FCM models for occupation-specific costs, macroeconomic conditions, vacancy chains and disability conditions. We used longitudinal registered data of real-life stroke patients followed up until the age of 50 years with linkage to nationwide registers of their sickness benefits, mortality and earnings. The objective individual-level data were obtained from a large, unselected, population-based birth cohort, representative of the general Finnish population living in a Northern European welfare society. As a strength of this study design, the productivity cost estimates were compared between different stroke types, occupational and educational classes, and between previously employed and non-employed at the time of stroke.

Importantly, since sickness absence for stroke is usually prescribed for at least two weeks, even in the mildest cases to consider the risk of recurrent attack during that period, the accuracy of the registered data is high. A specific methodological strength indeed stems from the large availability of relevant and reliable Finnish register-based data. Especially, population-level data on occupational-specific vacancy durations and labour market conditions used in this study are unique within international literature. The validity and reliability of Finnish nationwide registers have been proved to be excellent [60, 61]. Furthermore, there are only few studies that have applied FCM with economic evaluations in countries outside the Netherlands [15].

This study has also limitations. The numbers of cases in stroke subtypes were small and inclusion of a control group was not possible in this analysis. The register data did not include information on the stroke severity or recurrence, symptoms, lesion size, lesion location, or given treatments. Also, the data of SII only included the dates of sick leaves longer than 10 days. Additionally, a previous study in Finland has shown that people not currently working because of unemployment or studying do not receive sickness allowance or disability pension, to which they are similarly entitled to, as easily as employed people [62]. If this were the case, the rate of disabled stroke patients could be actually higher than was found, as some disabled stroke patients are not present in registers. However, the productivity cost estimates of previously unemployed stroke patients were as high or even higher than of those working at the time of stroke event.

### Implications

This study compared different models estimating productivity costs. Our results can be used for further development of pharmacoeconomic guidelines regarding productivity costs. Whenever data are available, we recommend the use of adjusted models of HCM and FCM as they take into account the criticized hidden assumptions of the models and minimize the effects of possible biases. Adjusted models shown in this study are generic and therefore assumably generalizable to other diseases. However, the results of monetary amounts are disease specific, as seen when comparing the productivity costs in different stroke subtypes. It is known that future capacity to work is associated with stroke severity and clinical recovery after a stroke [16]. Nevertheless, also pre-stroke demographic factors affect the probability to return to work after a stroke [53].

It should be noted that neither the HCM nor FCM model adjustments, i.e., future participation in labour force or vacancy chains, can be measured with one existing value. Future participation in labour force can, however, be estimated based on disability free life expectancy, unemployment rate and decline in work ability. Vacancy chain can be estimated based on unemployment rate and proportion of employed jobseekers. Our results provide empirical support for the adjusted approaches, as suggested in previous studies [62].

A previous study estimating societal costs of strokes in United Kingdom showed that among working aged individuals, total costs were £46,000 in first year and £26,000 in subsequent years [63]. Of these, HCM productivity costs accounted for £5,200 in first year and £5,900 in subsequent years. It can be assumed that with adjustments presented in our study, the productivity costs of the UK sample would have decreased 35%, and, therefore, the total costs would have decreased 4% and 8%, respectively. In economic evaluations such a difference in cost estimations due to bias in method assumptions can lead to erroneous decision making.

## Conclusion

This paper compared adjustments for human capital and friction cost methods in evaluating productivity costs of stroke. This study highlights the importance of adjustments, especially related to vacancy durations and macroeconomic conditions, and encourages future studies to make use of routine data to generate more accurate productivity estimates for acute health shocks.

## Supplementary Information

Below is the link to the electronic supplementary material.

Supplementary file1 (DOCX 23 KB)

## Data Availability

Data are available from the Northern Finland Birth Cohort (NFBC) for researchers who meet the criteria for accessing confidential data. Please, contact NFBC project center (NFBCprojectcenter@oulu.fi) and visit the cohort website (www.oulu.fi/nfbc or http://urn.fi/urn:nbn:fi:att:bc1e5408-980e-4a62-b899-43bec3755243) for more information.
