# Supplementary material for: Adjusted productivity costs of stroke by human capital and friction cost methods: a Northern Finland Birth Cohort 1966 study
Source: Eur J Health Econ. 2021 Feb 24;22(4):531–45. doi: 10.1007/s10198-021-01271-7 (PMC8166714; doi:10.1007/s10198-021-01271-7)
Supplement: Supplementary file 1 — Supplementary file1 (DOCX 23 KB) [file 10198_2021_1271_MOESM1_ESM.docx]

*Supplement*

The healthcare system of Finland is universal, publicly organized and publicly funded. The complementary private healthcare services constitute a minor share of services and make statutory reports to the same national databases as the public sector does. Especially concerning severe diseases, the main responsibility lies on the public health care sector. Permanent residents of Finland are registered for social security benefits, as sickness insurance of Social Insurance Institution (SII) and regarding employment-related data and benefits in the Finnish Center of Pensions (FCP).

According to legislation of Finland, the employer is responsible for paying an employee full wage for the first 10 weekdays (Mondays to Saturdays) of sickness absence [1]. In work contracts, however, longer periods of full sickness pay can be agreed upon, often covering 1–2 months. Sickness allowance is paid after the initial 10 days’ employers’ deductible period by SII [2]. In cases of paying full wage for longer sickness periods, the employer receives the corresponding allowance. For farmers the deductible time is four days and for other entrepreneurs one day. In cases of accidents, the insurers start paying sickness allowance the next day in cases where the sick leave exceeds 3 days. Partial sickness allowance was launched in 2007 and can nowadays be granted for a maximum of 120 days. The maximum duration for full sickness allowance is 1 year, whereafter full or partial disability pension can be granted, either for fixed term (called rehabilitation subsidy) or permanently [3]. Resulting from the varying deductible times before sickness allowances are granted, the shortest sickness absences are not uniformly registered, whereas all sickness absences longer than 10 days and all pensions are comprehensively covered in the registers of SII and FCP.

The Finnish Tax Administration (FTA) collects taxes from individuals, business owners and self-employed people. In Finland, taxes are paid both on earned income and capital income. Earned income consists pays from employment, pensions and social benefits.

*References*

1. Finlex. In:  Employment Contracts Act. <https://www.finlex.fi/en/laki/kaannokset/2001/en20010055.pdf>. Accessed Apr 17th 2020.

2. Social Insurance Institute of Finland. In: Sickness allowances. <https://www.kela.fi/web/en/sickness-allowances>. Accessed Apr 17 20120.

3. Finnish Centre for Pensions. In: Pensions part of social security. <https://www.etk.fi/en/the-pension-system/pension-security/pension-as-social-security/>. Accessed Apr 17th 2020.

stylefix
